# Applications of Medical Tattooing: A Systematic Review of Patient Satisfaction Outcomes and Emerging Trends

**DOI:** 10.1093/asjof/ojab015

**Published:** 2021-05-04

**Authors:** Stacie J Becker, Jeffrey E Cassisi

**Affiliations:** University of Central Florida, Orlando, FL, USA; Department of Psychology, University of Central Florida, Orlando, FL, USA

## Abstract

**Background:**

Medical tattooing is often applied in the context of plastic, aesthetic, and reconstructive surgery to help achieve the best cosmetic outcome.

**Objectives:**

This article reviews various conditions that medical tattooing has been empirically studied in terms of patient satisfaction outcomes, makes practice recommendations, and suggests future directions for research.

**Methods:**

This review was performed following the PRISMA guidelines. Studies were included if the tattooing application was associated with a medical condition and if outcome data were provided using at least a case series methodology. Where no cohort or clinical series exist, case examples are used from the literature and the author’s practice to illustrate emerging medical tattooing applications that need further evaluation.

**Results:**

Eighteen studies met the inclusion criteria and addressed the following conditions: baldness, vitiligo, scars from incisions, lacerations or burns, and nipple-areola complex reconstruction.

**Conclusions:**

The application of medical tattooing has shown high levels of patient satisfaction across conditions. The practice recommendation grade is “B” or recommend since the level of evidence for these interventions ranged from III to IV according to the American Society of Plastic Surgeons guidelines. This means clinicians can consider this treatment alternative, but they should be alert to new information and be sensitive to patient preferences. Recommendations are made for reporting future research including clearly describing procedural details, identifying the professional performing the procedure, increased use of standardized outcome measures, and that satisfaction ratings be assessed by someone independent of the health service provider. Further research using randomized controlled trial methodology with waitlist controls is needed.

**Level of Evidence: 4:**

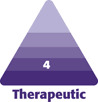

Tattooing is defined as the introduction of exogenous pigments into the dermis, resulting in a permanent mark. Tattooing has been most often used for decoration; although, it is being increasingly used with many medical conditions. This paper reviews the patient satisfaction outcomes when tattooing is used in a restorative and reconstructive medical context. The application of tattooing in these situations is herein referred to as medical tattooing. Medical tattooing is applied to several disorders and is used in a variety of cosmetic and reconstructive applications such as following radical mastectomies in patients with cancer.

## METHODS

**Figure 1.**
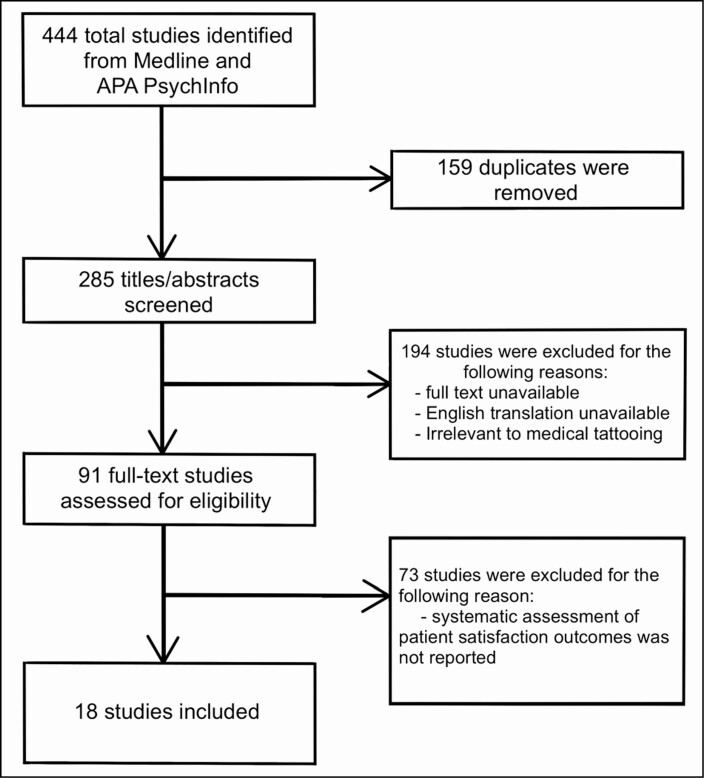
The flow of scoping review and reasons for study exclusion.

The PRISMA extension for Scoping reviews was followed to conduct this systematic review (Appendix).^1^ The keywords and medical subject headings that were used to search peer-reviewed studies within 2 databases (Medline and APA PsychInfo) are reported in Table 1. The steps taken to identify the studies included in the review are presented in a flowchart in Figure 1. The first author (S.B.) screened each study for relevance based on title and abstract. The full text of each study was then reviewed by both authors (S.B. and J.C.) to verify inclusion or exclusion criteria. The review was conducted on existing published research through September 2020, and institutional review board approval was not required.

**Table 1.** Keywords and Medical Subject Headings Used in the Search of 2 Databases (Medline and APA PsychInfo)

Databases	Search terms
Medline and APA PsychInfo	tattooing AND patient satisfaction; dermatography; micropigmentation; dermopigmentation; medical tattooing AND plastic surgery; medical tattooing AND reconstruction; medical tattooing AND satisfaction; medical tattooing AND scar; medical tattooing AND alopecia; medical tattooing AND burns; medical tattooing AND vitiligo

Studies were included if they involved medical tattooing of the derma and reported systematic assessment of patient satisfaction outcome data. Studies were excluded if the medical tattooing was internal or used as markers for surgical or radiological intervention, if English translation was not available, and if patient satisfaction outcomes were not reported.

The primary empirical evidence used to evaluate outcomes was the overall patient satisfaction based on the percentages satisfied in each study. In addition, secondary outcomes were reported such as qualitative observations and rates of reported complications. Unfortunately, most of the studies failed to use a standardized or universal measure of patient-reported satisfaction which presented a challenge to aggregating data.

## RESULTS

The initial literature search based on the terms listed in Table 1 resulted in 285 unique peer-reviewed articles. A total of 194 of those articles were deemed irrelevant, because the full text could not be obtained, or English translation was unavailable. The remaining 91 full-text studies were assessed for eligibility and 18 of those studies were deemed eligible after meeting the inclusion criteria. Details for each study, including outcomes, are presented in Table 2. The average patient satisfaction ratings for physician-led restorative tattoos when reported was 92.2%. The average patient satisfaction ratings for non-physician tattoo providers when reported was 93.6%. Patient satisfaction ratings across all studies and conditions averaged 91.7%. 

**Table 2.** Patient Satisfaction Outcomes and Complications Listed by Study

Reference	Condition	Tattoo service provider	No. of patients	Average follow-up period	Patient satisfaction	Complications
Traquina^2^	Scalp	Physician	62	1.5 mo	100% overall satisfaction	Frontal edema (1)
Park et al^3^	Scalp	Physician	80	6 mo	96% overall satisfaction	None reported
Mahajan et al^4^	Vitiligo	Physician (assumed)	30	6 mo	83.4% overall satisfaction	None reported
Ju et al^5^	Vitiligo	Physician (assumed)	14	1 mo	96% overall satisfaction	Erythema and swelling
Francis et al^6^	Vitiligo	Physician	30	75 mo	100% overall satisfaction	None
Guyuron and Vaughan^7^	Scars	Physician	18	17.39 mo	78% overall satisfaction	Initial hyperpigmentation
Drost et al^8^	Scars	Physician	56	Unknown	78% overall satisfaction	None reported
Yeates et al^9^	Scars	Medical tattoo practitioner	25	Unknown	88% overall satisfaction	None reported
Spear and Arias^10^	NAC	Physician and nurse	103	25.2 mo	83% overall satisfaction	Infection (5) Rash (1) Slough (1)
El-Ali et al^11^	NAC	Surgeon	40	14 mo	90% overall satisfaction	Infection (1) Slight fading an average of 32% (37)
Clarkson et al^12^	NAC	Nurse	36	Unknown	97% overall satisfaction	Rash (1)
Potter et al^13^	NAC	Nurse	14	3 mo	100% overall satisfaction	Slight fading (5)
Goh et al^14^	NAC	Nurse	110	38.5 mo	88% overall satisfaction	Minor dressing reaction Erythema
Liliav et al^15^	NAC	Surgeon (assumed)	29	8 mo	100% overall satisfaction	Irregular dye uptake
Smallman et al^16^	NAC	Surgeon and nurse	93	0.5 mo	93% overall satisfaction	None reported
Uhlmann et al^17^	NAC	PhD	20	Unknown	95% overall satisfaction	None reported
Cha et al^18^	NAC	Surgeon	95	6 mo	81% overall satisfaction	None reported
Starnoni et al^19^	NAC	Plastic surgery residents	48	12 mo	92% overall satisfaction	Scar dehiscence (3)

NAC, nipple-areola complex.

### Applications of Medical Tattooing

Typically, medical tattooing is applied with 1 of the 2 goals in mind: (1) Restoration, adding pigment to restore the appearance of the dermis so that it approximates its premorbid state and blends in with the surrounding skin, or (2) Camouflage, adding an aesthetically pleasing artistic image or pattern to conceal surface defects or blemishes. Both applications of tattooing have cosmetic and restorative goals in a medical context. This is not to be confused by how the term “cosmetic” may be used in a nonmedical or traditional tattoo studio as permanent “make-up.” In the latter case, the term cosmetic may refer to the tattooed application of eyeliner and lipliner. Medical tattooing does not focus on these applications but instead concentrates on a variety of restorative solutions for conditions, such as baldness, vitiligo, and scars.

#### Scalp Tattooing

A common medical tattoo application is to add pigment to the scalp in cases of advanced male pattern baldness and alopecia. The goal of medical tattooing of the scalp is to provide the illusion of the appearance of hair follicles in areas with hair loss, including scars. Two studies were identified which describe this application and provide meaningful outcome data.^2,3^

**Figure 2.**
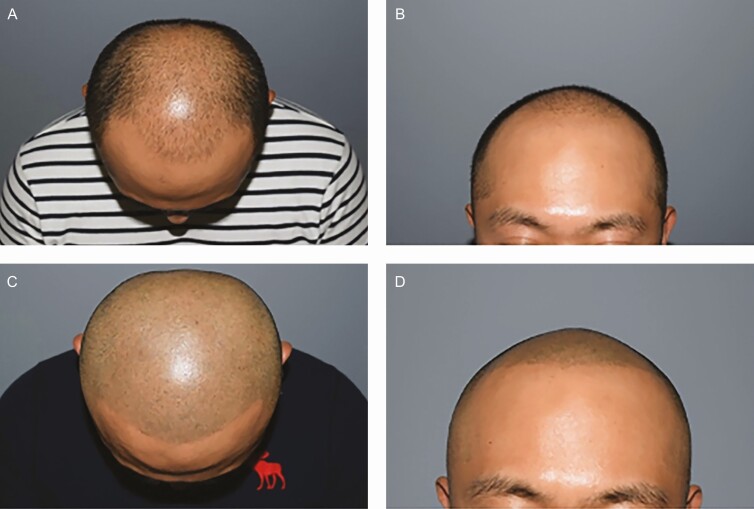
Shaved hair-style scalp tattoo on a 34-year-old male with Norwood Stage 5 alopecia. (A) Before top view, (B) before front view, (C) after top view, and (D) after front view. Photographs courtesy of Park et al.^3^

A recent illustrative study by Park et al published in 2019 treated and observed 80 patients for over 6 months with what the authors termed “scalp medical tattooing”.^3^ Their technique applies a “shaved hairstyle” scalp tattoo (Figure 2) performed by a physician. Of the 80 patients, 26 of them previously had at least 1 hair transplant surgery prior to the medical scalp tattoo treatments. The number of scalp tattoo treatments performed ranged from 2 to 7 treatments. Patient satisfaction in this study was an average of 4.8 (out of 5 on a Likert Scale with 5 being the highest possible). Results were evaluated by 2 expert hair transplant surgeons who were not involved in the scalp tattoo procedure and averaged 4.7.^3^

These 2 articles support the use of medical tattooing of the scalp in cases where baldness patterns are stable and if hair transplant options are not available or exhausted.^2,3^ Medical tattooing in both cases was performed by physicians, at a hair transplant clinic in the first case and a plastic surgery clinic in the second. The Traquina article provides details for the procedure.^2^ The critical issue for success is choosing the right color to match with the hair. Medical tattooing of the scalp is not recommended for patients with very active and progressive hair loss.

Narrow scars due to incisions from neurosurgery and from facial reconstructive flaps that extend to the scalp area may be more visible when hair growth does not return or cover the area. Gustavo Londoño, MD, a plastic surgeon with a long history of incorporating medical tattooing in his practice from Bogota, Columbia, reported in a personal communication successfully camouflaging these scars by tattooing the hairless hypopigmented area to match the surrounding hair color (instead of matching skin color). We are not aware of any systematic study of this application of scalp tattooing. Satisfaction with the results may vary with the width of the scar and other factors which need to be studied systemically. Nonetheless, this application may prove to have a large market.

The use of scalp tattooing as an adjunct to other medical treatments of baldness such as Minoxidil (Rogaine) is also an area of future study. However, many variables need additional evaluation. For example, what happens as the patient ages and hair color changes; what is the best cleaning solution to use; and do patients tolerate the procedure with topical anesthetics or nerve blocks? These questions can have an impact on the pigmentation appearance and durability. Edema has been reported in a few cases but resolved within days. Reliable indicators of when edema needs medical attention need to be standardized.

#### Vitiligo

Vitiligo is a medical condition in which the cells that produce melanin stop functioning, causing the skin to lose pigment, turning white. Vitiligo occurs in approximately 1% of the population. This skin disorder affects all races, although it is more noticeable in darker pigmented skin because of the contrast between the affected area and unaffected areas. The goal of micropigmentation in patients with vitiligo is the restoration of the appearance of skin color. That is to add pigment to blend the affected areas in with the surrounding skin tone. The challenge is to achieve the best pigment color match as possible. Three studies were identified which describe medical tattooing procedures for vitiligo and provide successful outcome data.^4-6^

**Figure 3.**
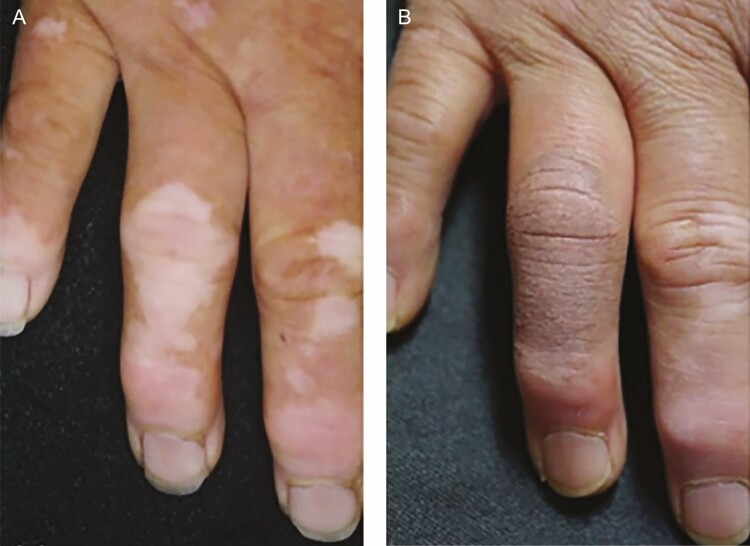
Vitiligo of the fingers on a 56-year-old female (A) before the procedure and (B) just after the procedure. Photographs from Ju et al.^5^

**Figure 4.**
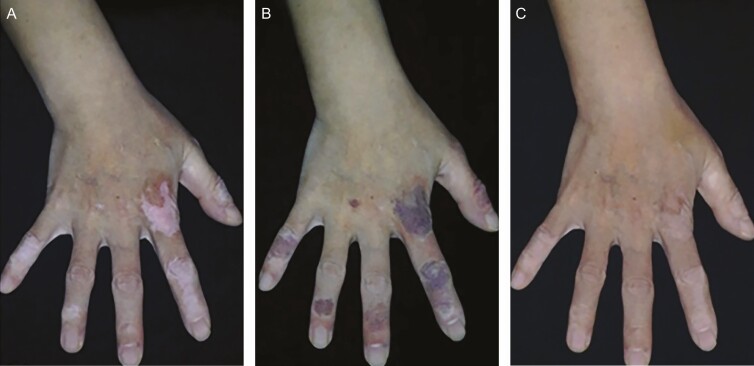
Vitiligo of the hand dorsum and fingers on a 49-year-old female (A) before the procedure, (B) immediately after the second procedure, and (C) 12 months after the final procedure. Photographs from Ju et al.^5^

**Figure 5.**
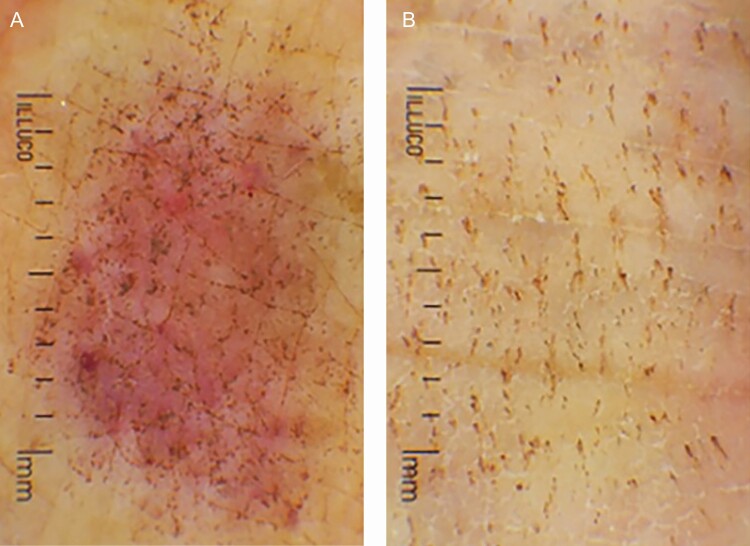
Dermoscopic findings on a tattooed hand dorsum of a 67-year-old female (A) immediately after the procedure and (B) 1 week after the procedure. Photographs from Ju et al.^5^

**Figure 6.**
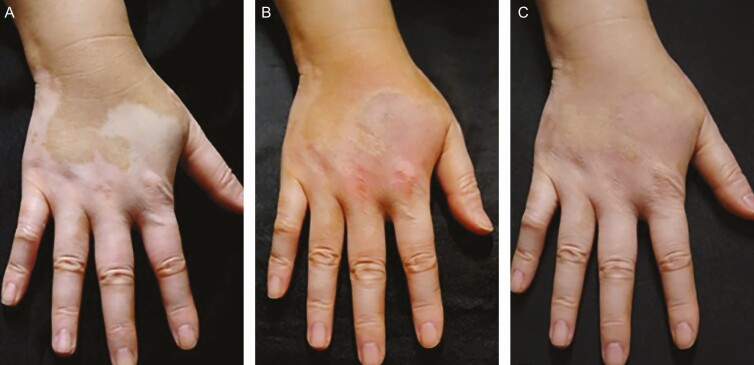
Vitiligo of the hand dorsum treated with gradation technique on a 46-year-old female (A) before the procedure, (B) immediately after the second procedure, and (C) 1 month after the final procedure. Photographs from Ju et al.^5^

A recent study by Ju et al published in 2020 reported on the application of medical tattooing to treat vitiligo patches in 14 patients with a total of 25 patches being tattooed.^5^ The person responsible for performing the tattooing was not identified. All patients had light to moderately colored skin. The procedure began by cleaning the vitiligo patches and the application of local anesthesia. The sharp margins of affected areas were blurred by applying a gradual color change during the tattoo session. Pigment selection and repeating the procedure were crucial for satisfactory color matching. Physicians rated 80% of the treated patches as achieving excellent color matching. Patients’ satisfaction ratings were satisfied or highly satisfied in 96% of the cases. Patients reported the pain level during the procedure as moderate, although local anesthetic injection was not required. No allergic reactions were noted, and no Koebnerization or vitiligo reactivation was reported.^5^ Examples are displayed in Figures 3-6.

The outcomes of the vitiligo studies were positive with high levels of reported patient satisfaction and no adverse effects.^4-6^ Therefore, medical tattooing is a promising alternative for patients with vitiligo who have not responded to conventional medical treatments. These 3 studies highlight the importance of color and pigment selection to achieve the best outcome.^4-6^ An experienced medical tattoo specialist may be used to train physicians and other providers in attaining these skills. The type of evidence for these 3 studies is level III (cohort studies).^20^

#### Scar Restoration (Pigmentation Blending)

Medical tattooing can be utilized in the treatment of hypopigmented scars when the scars are not appropriate for surgical scar revision and cannot be significantly improved by laser treatment. The goal is restorative, that is, to add pigment to a scar that has lost color to have it blend in with the surrounding skin. Three studies were found that used this technique and included patient satisfaction outcomes.^7-9^

The study by Yeates et al published in 2018 described that burn scar restoration of the face is a good example.^9^ This was a retrospective online survey with patients who received referral to a “tattoo provider” for medical tattooing of hypopigmented facial burn scars. These referrals were made by a charitable foundation focused on comprehensive burn patient care in the United Kingdom. These medical tattoos included lip restoration, eyebrow restoration, and the blending of hypopigmented scars to the surrounding skin tone. Of the 35 patients who had received medical tattoo restorative procedures, 25 (71%) agreed to participate. The comprehensive assessment covered 8 areas: satisfaction, staff perception, general Quality of Life (QoL), mood, confidence, facial appearance, functional, ability, and relationships with others. The authors reported that 22 (88%) patients being satisfied with the results of their medical tattooing procedure. Self-confidence was reported as improved in 22 (88%) patients, mood was reported as improved in 19 (76%) patients, and the ability to socialize was reported as improved in 19 (76%) patients.^9^ This study, while retrospective, represents the most complete assessment of patient-reported outcome measurement by including psychological functioning and QoL.

These 3 studies have shown the utility of medical tattooing for restoring the appearance of the scarred dermis to its premorbid state especially in conditions of hypopigmentation.^7-9^ The Drost et al article is recommended for those with interest in viewing photographic examples of what is possible with this intervention for patients with head and neck cancer.^8^ The treatment of hypopigmented scars through medical tattooing received high ratings of patient and physician satisfaction. Recent studies have integrated other professionals into the provision of medical tattoos. In addition, these studies provided more details on the systematic assessment of outcomes. Taken together, these studies suggest that, once a maximum recovery from medical intervention has been achieved, medical tattooing may further improve cosmetic outcomes. The type of evidence provided by these 3 studies is Level III (cohort studies).^20^

#### Scar Concealment

In cases of scars being hyperpigmented, having an uneven texture, or covering a large area, a restoration approach is not likely to produce a satisfactory outcome. In these cases, the goal of medical tattooing is to conceal the scar with an aesthetically appealing image that either covers it or camouflages it. In 2009, Spyropoulou and Fatah were one of the first to describe decorative scar concealment as an application of medical tattooing.^21^ Several cases were referred to a “tattoo artist” from a Plastic and Reconstructive Surgery Clinic, and the outcomes were photographically documented; however, patient satisfaction was informally described as “delighted” or “liked it.” ^21^ We believe that decorative scar concealment is one of the most common applications of medical tattooing in a cosmetic or reconstructive context, yet surprisingly few empirical studies evaluating patient satisfaction outcomes were found. This may be due to the fact that scar concealment is often patient initiated and not prescribed by physicians. Hence, systematic empirical study of outcomes has not been conducted. The type of evidence is level IV for this case series.^20^

**Figure 7.**
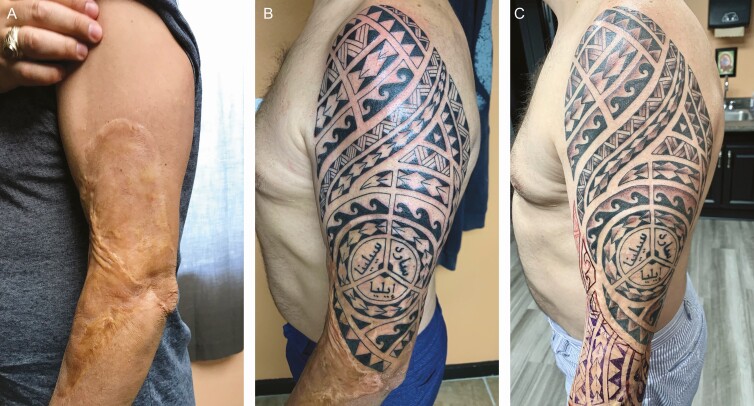
A case study using medical tattooing to conceal a large burn scar on the arm of a 48-year-old male who received a burn scar in childhood. (A) Mature burn scar, (B) first-phase scar concealment, and (C) second-phase embellishments.

**Figure 8.**
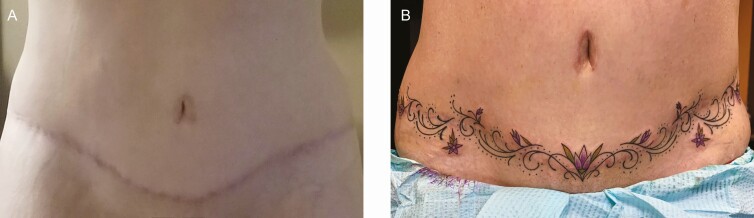
A case study using medical tattooing to conceal an abdominoplasty scar on a 49-year-old female (A) before and (B) after tattooing.

To illustrate the potential of this approach, we present 2 unpublished case studies of scar concealment with decorative tattoos from our clinic. The first case involves a large burn scar on the arm (Figure 7), and the second covers a long incision on the belly associated with an abdominoplasty (Figure 8). Both individuals were asked to rate their satisfaction with the tattoo as part of standard patient care to track the effectiveness of services. The patient with the burn scar on the arm reported being extremely dissatisfied with the appearance of the scar prior to tattooing and reported being extremely satisfied with the appearance of the burn scar post-tattoo. The patient with the abdominoplasty reported being somewhat dissatisfied with the appearance of the scar prior to the tattoo and reported being extremely satisfied with the appearance of the scar post-tattoo. Both patients reported being extremely satisfied with the overall tattoo procedure. Clearly, larger-scale empirical research of patient-reported outcomes is needed in this area.

### Three-Dimensional Restorative Tattooing

Three-dimensional (3D) restorative tattooing has the goal of providing an illusion of the premorbid appearance of the dermis. The skill and experience level required to execute 3D restorative tattoos are much higher than just adding color to the pigment. Matching skin tones is the basis of restorative treatment for conditions of hypopigmentation, but it is expanded on in 3D tattooing by utilizing light and shadow with pigments to create the illusion of depth in the dermis where there is none. The most commonly studied restorative tattoos have been with patients with a history of breast cancer needing nipple-areola complex (NAC) reconstruction tattoos.

#### NAC Restorative Tattoos

The treatment of breast cancer often requires surgeries, which can result in the removal of one or both NACs. Medical tattooing offers a unique solution to the issue of the missing NAC, either in combination with different nipple reconstruction techniques or as a “tattoo only” option by creating a 3D image in the place of a reconstructed nipple. Ten studies were found that evaluated patient satisfaction with the application of NAC tattooing.^10-19^

A recent study exemplifying the application of NAC restorative tattoos was by Starnoni et al in 2020.^19^ Forty-eight patients who underwent NAC restorative tattoos 3–8 months after the nipple reconstruction was completed by plastic surgery residents at Modena University Hospital in Italy. Patient satisfaction data were recorded at a 12-month follow-up appointment. High rates of satisfaction were shown with 67% reporting being very satisfied, 25% being satisfied, and 8% were reported as being dissatisfied with the results. The study concluded that “…[tattooing] should be performed by well-qualified healthcare workers under the supervision of a physician.” ^19^ All of the 10 studies show high patient satisfaction, and a study by Cha et al in 2020 suggested that a tattoo-only method had higher patient satisfaction than other NAC reconstruction methods.^18^ However, caution is advised in overinterpreting this result because the severity of disease was not controlled for in this study, and patients requiring surgery were likely to have worse disease requiring more disfiguring interventions. Nonetheless, NAC tattooing has minimal complications and does not require the convalescence that nipple reconstruction surgery does. 

The studies reviewed all involved breast reconstruction following cancer treatment. The application of 3D NAC tattooing is likely to be an equally successful intervention in cases where nipples have been disfigured for other reasons. For example, nipple necrosis is a rare side effect of breast augmentation, lift, and reduction surgeries. No systematic evaluation of this use of NAC restoration was found although we have encountered it in our practice.

A trend of increasing reliance on nonphysicians to provide NAC reconstruction is appearing in the literature. This trend of relying on professional tattoo artists and nurses was first acknowledged in an article by Ho-Asjoe and Mallucci in an editorial forum published in 2004.^22^ Fourie and Bruce-Chwatt responded by providing procedural details so that surgeons can satisfactorily perform NAC tattooing.^23^ This issue has continued to be discussed in the literature by Carney et al^24^ and Halvorson et al.^25^

**Figure 9.**
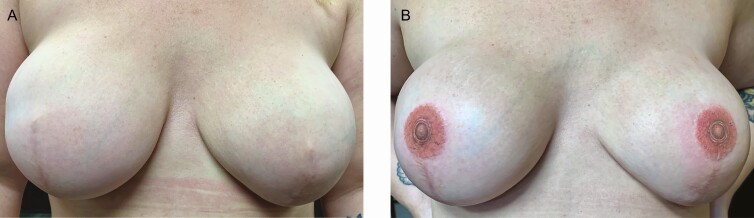
A case study of a 33-year-old female using 3-dimensional tattooing for nipple-areola complex restoration following mastectomy and breast reconstruction (A) before and (B) after tattooing.

**Figure 10.**
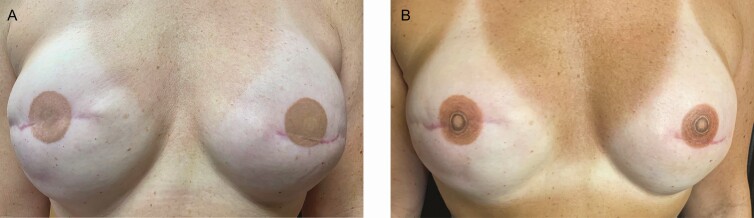
A case study using 3-dimensional tattooing to enhance a previously conducted 2-dimensional nipple-areola complex tattoo in a 46-year-old female. (A) Tattoo performed by a physician assistant in the medical office using a surface abrasion technique and (B) enhancement by experienced medical tattoo assistant applying the ink using dermal penetration.

**Figure 11.**
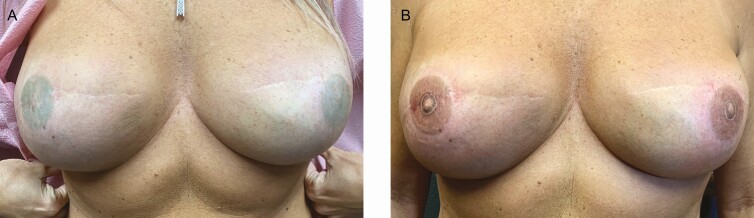
A case study using 3-dimensional tattooing to enhance a previously conducted 2-dimensional nipple-areola complex tattoo in a 47-year-old female. (A) Tattoo performed by a physician assistant in the medical office and (B) after enhancement by an experienced medical tattoo assistant.

The level of evidence presented in all 10 of the reviewed NAC studies is level III. Several case examples of NAC tattooing from the author’s practice are illustrated in Figures 9-11. All clients reported high satisfaction with outcomes.

**Figure 12.**
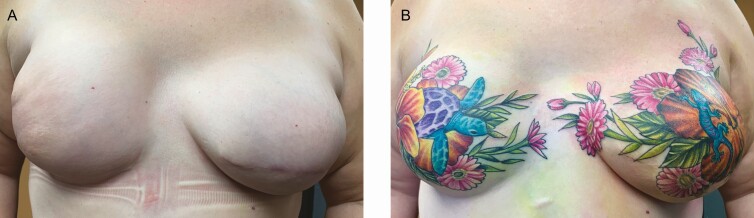
A case study of a 49-year-old female following 2 lumpectomies, and post-radiation treatment utilizing decorative scar concealment. (A) Before decorative scar concealment and (B) after decorative scar concealment.

Not all patients interested in tattooing of the breasts following medical intervention request NAC restorative procedures. In some cases, the patient is interested in using artistic tattooing to camouflage scarring and distortions to the breast contours. To quote one patient, “adding NAC tattoos is not going to make my breasts look exactly like they did before, and I’d rather go in a different direction.” The patient illustrated in Figure 12 was very satisfied with this approach.

Other applications of 3D medical tattooing are emerging. For example, our search also revealed one case study of medical tattooing used with syndactyly and also one case study of medical tattooing utilized in toenail bed restoration. These single case studies were not included in our estimates of average patient satisfaction, but they illustrate that applications of 3D medical tattooing outside of NAC reconstruction are emerging. More applications will likely present themselves over time.

#### Toenail Restorative Tattoo

**Figure 13.**
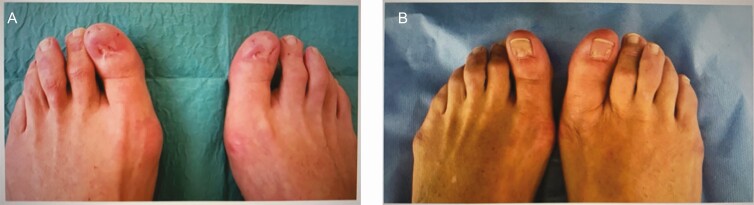
A case study using tattooing to restore the appearance of toenails in a 50-year-old female. (A) Toenail deformity caused by bilateral surgical avulsion procedure to correct onychocryptosis hallux and (B) post-surgery 3-dimensional tattooing restoring the appearance of toenails. Reproduced with kind permission from Renzoni et al.^26^

A case study published in 2017 presents a patient who previously underwent a bilateral surgical avulsion procedure to correct Onychocryptosis hallux of both of her big toes.^26^ The procedure resulted in the loss of her toenails on these toes. The missing toenails led the patient to experience psychological discomfort and loss of self-esteem.^26^ The recommendation of medical tattooing to restore the appearances of the nail beds of the big toes was made by the patient’s physician, who also supervised the tattoo artist during the procedure. The shape and color of the toenail were discussed and agreed upon with the patient beforehand. The artistic use of shadows and highlights created a realistic appearance of the nail beds as shown in Figure 13. This study also stressed the importance of the training for a tattoo artist performing medical tattoos and recommended a knowledge of anatomy and skin pathology, a thorough knowledge of the tattooing equipment and materials, and the implementation of correct procedures and hygiene rules.^26^ The patient expressed full satisfaction with the results and felt the procedure enabled her to reestablish a sense of physical integrity. Further research exploring the impact of medical and restorative tattoos on self-esteem is a sorely needed area of research. The level of evidence in this study is rated level IV (case study).^20^

#### Syndactyly

Medical tattooing has also found utility as a simple alternative treatment for syndactyly of the toe. Syndactyly is characterized by fusion or webbing between 2 or more digits.^27^ Simple syndactyly is involving soft tissue only, whereas complex syndactyly involves bony union. If the fusion of the digits travels to the tip of the digit, it is referred to as complete syndactyly and incomplete if it does not. This condition in any form can cause psychological and emotional distress for the sufferer. Patients might request surgical treatment of both simple and complex syndactyly because of this distress. Simple syndactyly does not often require the intervention for functional purposes. Several surgical techniques can be utilized. Surgery always carries the risk of infection, neurovascular damage, joint contracture, skin graft failure, donor site morbidity, scarring, keloid formation, or a progressive distal migration of the web or “web-creep.” ^27^ The case shown in this 2016 study included a patient with incomplete simple syndactyly who presented for hallux valgus correction surgery. Surgery was prompted by the patient’s anxiety about the appearance of her webbed toes. Medical tattooing was suggested as a possible treatment, and after a consultation with the tattoo artist, the patient proceeded with the tattoo procedure instead of surgery. The patient reported being very satisfied with the outcome.^27^ Clearly, more studies are needed using more patients to fully evaluate this application of medical tattooing. The level of evidence provided for this study is level IV (case study).^20^

## DISCUSSION

The various conditions systematically treated with medical tattooing are listed in Table 2. Approximately, 900 patients across these studies utilized medical tattooing as an intervention. Most of the early studies describe the application of medical tattooing performed by a physician specialist. These studies reported restorative applications to a flat level area to effect color matching with the surrounding tissue and high levels of patient satisfaction were found. Recently, there has been an increase in the studies using medical tattooing to produce 3D effects. The most common application of medical tattooing in this context is NAC restoration, but other applications of 3D tattooing are emerging. Associated with this trend is the increased use of Nurses and Medical Tattoo Assistants to perform these procedures. This trend is logical as patients’ expectations increase for more aesthetic and artistic outcomes. The development of artistic nuances in medical tattooing requires training and time that may not be cost effective for many physicians, whereas these nuanced skills are the focus of specialized Medical Tattoo Assistant who primarily comes to the field after gaining extensive experience from a purely artistic perspective. 

Potential limitations of this study are affected by the lack of standardized patient-reported outcomes and the limited access to data about techniques, equipment, and training of service providers. Outcome research can be significantly improved with better methodology and more procedural details. Based on this review, we suggest that future studies should clearly report the following in the Methods, Materials, Procedures, and/or Results sections:

1. Who conducted the tattooing (MD, nurse, or medical tattoo assistant).
2. Training and experience in tattooing of the MD, nurse, or medical tattoo assistant.
3. Number and duration of tattooing sessions required to complete procedure.
4. Equipment brand, standard settings, needles, and inks.
5. Standardized patient-reported outcomes and satisfaction.
6. Whether the patient-reported outcomes and satisfaction ratings were collected by the provider/proxy or independent assessor blind to the procedure.

In the future, outcomes may be more thoroughly evaluated if several other methodological steps are used such as:

1. Standardized measures of physician ratings of aesthetic outcome.
2. Standardized measures of independent ratings of aesthetic outcome.
3. Pre- and post-tattoo measures of body dysphoria.
4. Increased use of patient self-esteem and QoL measures to get a clearer picture of the potential psychological utility of medical tattooing.
5. Randomized controlled trials (RCTs) with waitlist controls.

### Patient Satisfaction

Overall, patients reported high satisfaction rates across all conditions that utilized medical tattooing as an intervention. The follow-up period varied within each category of condition as well across all conditions reviewed. The shortest follow-up period was an average of 2 weeks, and the longest average follow-up period was 38.5 months. The lowest average satisfaction rating was scar tattooing with 78% and the highest rating was 100% found in NAC tattooing, vitiligo tattooing, and scalp tattooing. See Table 2 for the overall patient satisfaction data listed per study.

### Complications and Future Directions

Complications were reported in some studies although most were reported as resolved without further intervention. The most common complications reported were erythema (irritation of the skin), edema, initial hyperpigmentation, and slight fading of the pigment. Other complications included minor dressing reaction, scar dehiscence, infection, rash, and slough (Table 2).

Of particular interest is the issue of tattoo fading. It is not clear why some tattoos are resilient to fading and others fade more rapidly. Generally, it is our experience that most of the tattoos will last 10 or so years before touch-up or additional treatments are required. We have encountered tattoos that fade more rapidly, and we postulate that sun exposure is the primary cause; however, this has not been empirically studied. The brand of ink used may vary in terms of resistance to fading. Unfortunately, existing studies do not specify which brand was used. We are aware of some proprietary inks being advertised which claim to be superior for medical tattooing. Such assertions about proprietary inks need to be evaluated in independent empirical trials, and if these inks are marketed for medical applications, they need to be evaluated by the Food and Drug Administration (FDA) for performance and safety.

Adequate information about the instrumentation, inks, person performing the tattooing, and techniques is rarely available. For example, it is our impression that many of the tattoos applied by physicians in the office sometimes use surface abrasion techniques, whereas those performed by medical tattoo assistants and other tattoo professionals use dermal penetration techniques. The relative effectiveness of these techniques needs to be empirically evaluated. It is our impression that surface abrasion techniques lead to greater scarring, which makes touch-ups and follow-up treatment more difficult.

The application of cultured melanocytes has been successfully used in the treatment of vitiligo.^28^ In addition, one recent article by Tsao et al in 2019 used surgically transplanted melanocytes with success in blending white scars with the surrounding skin.^29^ However, the application of cultured melanocytes using tattooing equipment is speculative at this time, and the costs of such an approach may outweigh the benefits. Such advances would necessitate the intervention being conducted in the dermatologists or plastic surgeon’s office.

This study contributes to the knowledge base of medical tattooing by organizing the conditions in which medical tattooing can be used as an intervention and shows a need for standardized outcome measures and techniques. This study reports on the existing outcome data for the field with a focus on patient satisfaction and also makes practice recommendations and suggestions for future research on the subject.

## CONCLUSIONS

Medical tattooing is applied but not limited to hair loss, vitiligo, scar concealment, finger and toenail appearance restoration, syndactyly, and the restoration of the NAC. Other uses for medical tattooing are likely to appear over time. All studies reviewed found that medical tattooing was associated with the overall patient satisfaction of 91.7% across all studies. Outcomes appeared equivalent whether the tattooing was performed by a physician, nurse, or a medical tattoo assistant. Benefits for the procedures outweighed reported adverse side effects and harms. Using the American Society of Plastic Surgeons (ASPS) Evidence-Based Clinical Practice Guideline Methodology, the level of evidence was based entirely on outcome studies, retrospective cohort studies, and case series with no RCTs.^20^ Since the level of evidence for these interventions ranged from III to IV according to the ASPS guidelines, the practice recommendation grade is “B” or recommend.^20^ This means clinicians can consider this treatment alternative, but they should be alert to new information and be sensitive to patient preferences.

We have argued elsewhere that the establishment of a health service profession called the Medical Tattoo Assistant closely integrated with Plastic and Reconstructive Surgery and Dermatology is needed.^30^ The Medical Tattoo Assistant is an evidence-based profession, and its establishment is necessary to protect the public, to assure quality and performance standards, a code of ethics, and to facilitate the accumulation and dissemination of specialized knowledge.^30^ Irrespective of which professional performs the procedure, a formal curriculum and systematic practical training experiences need to be developed to ensure the optimal outcomes for the patients receiving these interventions.

The area in need of the greatest future systematic study is the application of medical tattooing for patients interested in the concealment of scars. Surprisingly, few outcome studies focused on patient satisfaction for scar concealment were found even though we believe that this is one of the most widely used applications of medical tattooing.

## Supplementary Material

ojab015_suppl_Supplementary_Appendix
